# Supplementary material for: Effects of Graphene Derivatives and Near-Infrared Laser Irradiation on E. coli Biofilms and Stress Response Gene Expression
Source: Int J Mol Sci. 2025 May 15;26(10):4728. doi: 10.3390/ijms26104728 (PMC12112295; doi:10.3390/ijms26104728)
Supplement: Supplementary file 1 [file ijms-26-04728-s001.zip › ijms-3568939-supplementary.pdf]

Supplementary Information

Growth of *E. coli* RO91 (*rpoS* expression)

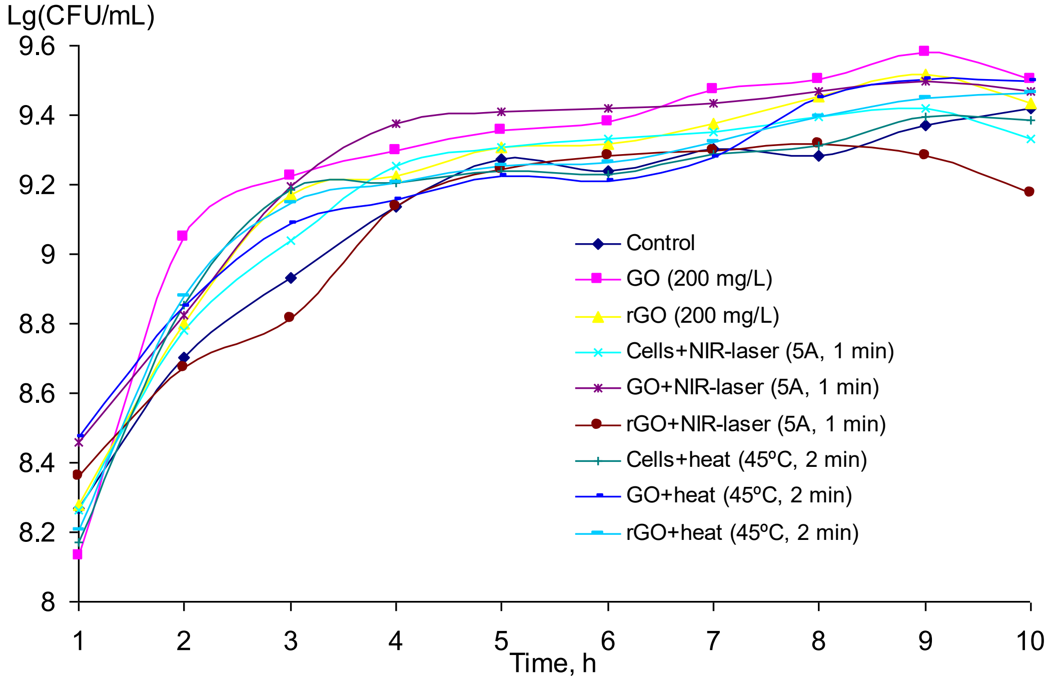

Figure S1. Decimal logarithm of CFU/mL of *E. coli* RO91 during growth in the presence of graphene derivatives and after NIR laser irradiation at 5A for 1 min and heating at 45ºC for 2 min

Growth of *E. coli* EH40 (*soxS* expression)

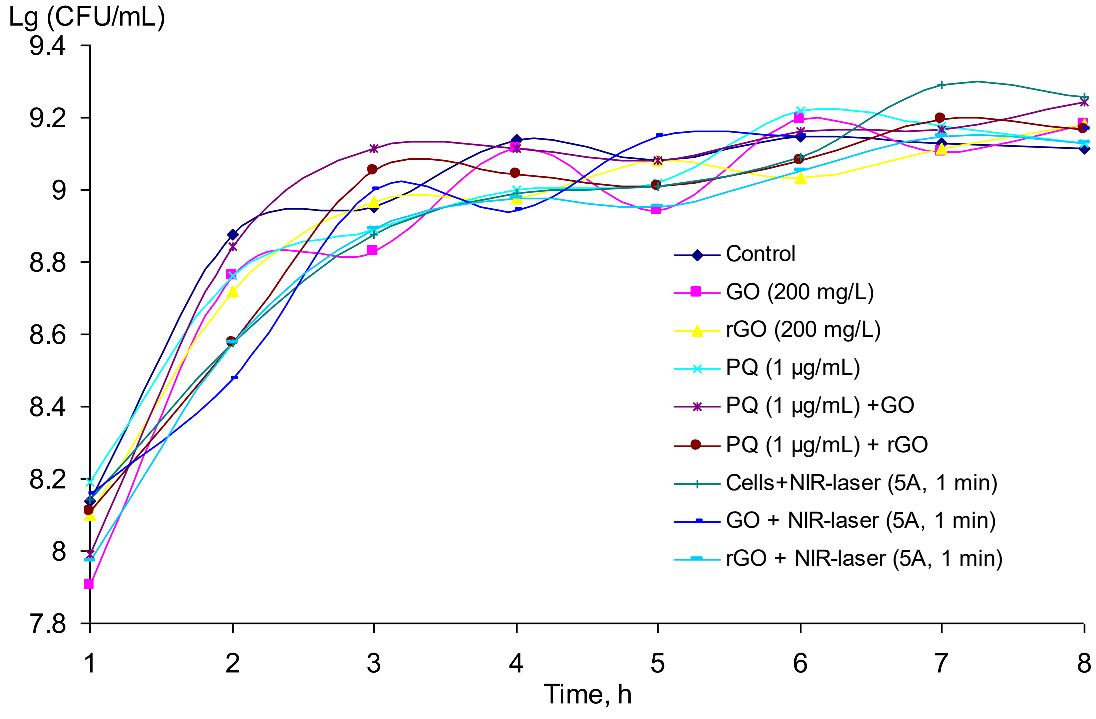

Figure S2. Decimal logarithm of CFU/mL of *E. coli* EH40 during growth in the presence of graphene derivatives and after NIR laser irradiation at 5A for 1 min

NIR laser

The first stage of the laser, which corresponds to a pair of Bragg gratings with reflection at a wavelength of 1200 nm, is pumped by radiation from an ytterbium fiber laser with a pump diode at 975 nm. The layout of the NIR laser is shown in Figure S3.

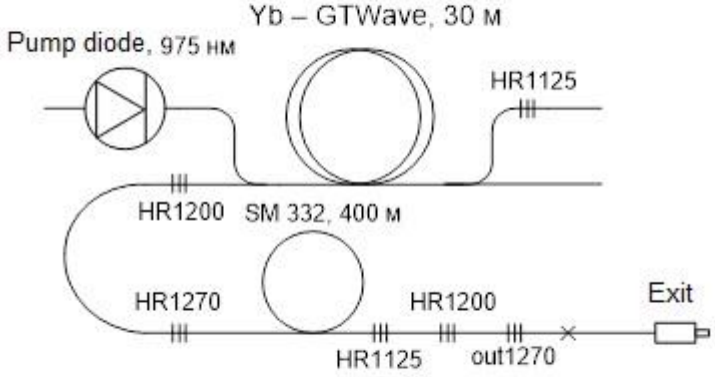

Figure S3. Schematic diagram of the NIR laser used in the work [38].
